# Predictors of the quality of the relationship between informal caregiver and care recipient in informal caregiving of older people: presentation and evaluation of a new item

**DOI:** 10.1186/s40359-024-01840-4

**Published:** 2024-06-11

**Authors:** Linda Becker, Elmar Graessel, Anna Pendergrass

**Affiliations:** 1 Department of Psychology, Chair of Health Psychology, Friedrich-Alexander-Universität Erlangen-Nürnberg (FAU), Nägelsbachstr. 49a, 91052 Erlangen, Germany; 2 Humanwissenschaftliche Fakultät, Vinzenz Pallotti University gGmbH, Vallendar, Germany; 3 Center for Health Service Research in Medicine, Department of Psychiatry and Psychotherapy, Universitätsklinikum Erlangen, Friedrich-Alexander-Universität Erlangen-Nürnberg (FAU), Erlangen, Germany

**Keywords:** Informal caregiving, Relationship quality, Geriatric care, Assessment tool, Caregiver burden, Caregiving stress, Caregiving motivation, Social desirability, Positive aspects

## Abstract

**Background:**

An important factor that has not been directly addressed very often in caregiver (CG) counseling to date is the quality of the relationship between the CG and the care recipient (CR). One reason is the lack of availability of a suitable assessment tool that is not strongly influenced by social desirability. Here, we present and evaluate a new item for the assessment of relationship quality (RQ) in the context of informal caregiving of older people.

**Methods:**

*N* = 962 informal caregivers of older people participated. Our item assessed RQ by providing three answer categories (positive, neutral, and negative) that were presented through the use of smiley faces. For evaluation, and to avoid bias due to social desirability, the neutral and negative categories were combined. We calculated a stepwise binary logistic regression.

**Results:**

Expected associations with the variables care burden, perceived positive aspects, and care motivation were found (all p values < 0.01). An exploratory analysis revealed that additional predictors of RQ consisted of the CR’s age as well as whether the CR’s diagnosis was dementia, CG’s amount of dysfunctional coping, and whether the CG was caring for more than one CR.

**Conclusions:**

We conclude that our item is well-suited for the assessment of RQ in the context of informal caregiving of older people. Because it uses language-free answer categories by means of smiley faces, our item can be applied easily. Bias due to social desirability can be minimized by dichotomization (i.e., combining the negative and neural answer categories). In future research, our tool should be evaluated in other contexts.

## Background

Numerous studies have shown that informal caregiving is associated with increased stress and depression and has a negative effect on the physical health and well-being of the informal caregiver (e.g., [1]). Nevertheless, in many cases, both the care recipient (CR) and the caregiver (CG) want the CR to be able to remain at home for as long as possible [2]. This situation also offers the best health-economic advantages [3]. Therefore, scientific research on the improvement of the support services for the CGs is essential. For example, new counseling concepts should be used not only to reduce negative aspects but also to expand and promote the positive aspects of caregiving [4, 5].

One important aspect of the informal care situation is the quality of the relationship between the CG and CR (relationship quality; RQ), which has not been directly addressed in CG counseling very often to date. Research has a long tradition to demonstrate the relevance of the quality of the CG-CR relationship and its impact on different aspects of the care situation [6]. One of the most important correlations is that between negative RQ and abusive behavior of the CG [7]. Negative relationships between CG and CR have been found to be associated with more abusive behavior of the CG than positive relationships. In addition, DeVito Dabbs et al. (2013; [8]) found that RQ is related to the self-help behavior of the CR and that a positive RQ can contribute to more positive self-reliance. In addition, it has been found that a positive RQ significantly reduces the likelihood that the CR will be placed in a nursing home [9]. In particular, studies have examined the impact of the relationship on the mental health of caregiving relatives. Lum et al. (2014; 10) as well as Tanji et al. (2008; 11) found that a positive relationship was associated with lower subjective burden and more positive aspects of the care situation in daily life and was negatively correlated with CGs’ depression. Lyonette and Yardley (2003; 12) also identified the quality of the relationship as a predictor of CGs’ satisfaction with care and subjective burden. Looking closely at the factor structure of subjective burden, the authors showed that the level of CGs’ satisfaction with the CG-CR relationship predicted the extent to which giving care directly impaired CGs’ lives and CGs’ frustration and feelings of shame [13]. Moreover, there is initial evidence that a positive RQ is associated with better physical health in CGs [14]. All these studies highlight the importance of conducting studies on the relationship quality between CG and CR in informal caregiving of older people, which is not only of scientific relevance, but also societally important, e.g., due to the reduction of the likelihood that the CR will be placed in a nursing home or the association between RQ and abusive behavior of the CG (see above).

Several scales have been established to evaluate relationships in couples [15–19], but none of them have been specifically designed for the context of informal caregiving. One of the best-known scales is the Relationship Assessment Scale [20], which measures relationship satisfaction with seven items. It has been validated in several languages and used in diverse contexts (e.g., [21, [22]). However, despite these validated scales, the quality of the relationship between the CG and CR has not been directly addressed very often in CG counseling to date [23, 24]. There could be numerous reasons for this lack of research. First, CGs usually do not indicate a negative relationship due to social desirability [25]. Second, a negative relationship is uncomfortable for CG counselors to address. Third, for economical and practical reasons, the existing scales are not suitable for use in the daily counseling setting. To address some of these challenges and promote the relevance of assessing the quality of the CG-CR relationship, we developed a new one-item assessment with intuitive, cross-cultural, and language-free answer options. This one-item approach makes our instrument easy and economically to use and, therefore, better suited for the setting of informal caregiving of older people than existing tools (see Methods for more information on our item).

The present study offers an evaluation of our new tool. If it is suitable for assessing RQ in informal geriatric care, known associations with care-related variables should be found. The first of these variables is CGs’ perceived burden. Several studies have shown that a negative relationship between the CG and CR is associated with more perceived care burden, stress, or overload [26–29]. The second factor includes positive aspects of the care situation (e.g., self-esteem, satisfaction), which have been shown to be related to a positive CG-CR relationship (e.g., [30–32]). The third factor is care motivation [33]. Intrinsic care motivation, such as caregiving because the CG is attached to the CR, has been shown to be related to positive RQ [34, 35]. By contrast, extrinsic care motivation (e.g., caregiving due to a sense of obligation) has been found to be associated with a negative CG-CR relationship and with perceived care burden and stress [12, 34].

### Aim of the present study

The main goal of the present study was to develop and evaluate a new RQ assessment tool that can be used in the context of informal geriatric care. Such an item is urgently needed due to the low number of existing studies on the RQ between CG and CR in informal caregiving of older people, for which one reason may be that to date no entirely suitable tool was available.

The idea behind our analyses was that if our item is suitable for assessing RQ in the context of informal caregiving of older people, known associations with typical predictors should be found. Our hypotheses were as follows:

1. RQ is negatively associated with perceived care burden.
2. RQ is positively associated with perceived positive aspects of the care situation (e.g., the feeling of being needed or of being important).
3. RQ is positively associated with intrinsic care motivation.
4. RQ is negatively associated with extrinsic care motivation.

Our second goal was to investigate whether RQ in informal caregiving of older people is associated with additional variables with regard to the CG (e.g., age, gender, employment status), the CR (e.g., age, gender, reason for the need for care), as well as the care situation (e.g., whether the CG and CR are living together). These variables were selected based on the variables and constructs usually collected in international research in the field of informal caregiving (e.g., [36]). See below for a detailed description of all variables.

## Methods

### Design and setting of the study

The study design was cross-sectional involving self-reported data. Data were collected in the context of the study “Benefits of Being a Caregiver” between October 2019 and March 2020 [37]. A total of 5,000 self-report questionnaires were distributed by 50 care assessors from the Medical Service of the Bavarian Health Insurance (MD) to statutorily insured informal CGs all over Bavaria (Germany). The MD Bavaria is the official consulting and expertizing service for the statutory health and nursing care insurance (SHI). The SHI is the standard national health care insurance and covers approximately 90% of the German population. The CGs applied for an initial grade or an increase in the CRs’ care level. The care level describes the extent to which care is needed on a 5-level ordinal scale: It is assessed by trained experts who are independent of the insurance system. Classification is based on the need for physical care and help in social and instrumental activities of daily living. Formal care is financed by long-term care insurance on the basis of the care level. Filling out the questionnaires had no influence on the application for an initial grade or an increased care level. The CGs filled out the questionnaires at their homes and sent them back via mail. By returning the completed questionnaire, 1,083 CGs (21.64%) provided informed consent. Six cases (*n* = 9) were removed because of missing information and *n* = 117 because the CRs’ age was younger than 65 years.

### Participant characteristics

The final sample included *N* = 962 informal CGs (62.0 ± 12.6 years, 24.4% male) who were involved in the informal caregiving of at least one geriatric person. A detailed overview of the sample characteristics is provided in Table 1. The study was approved by the local ethics committee of the Medical Faculty of the Friedrich-Alexander-Universität Erlangen-Nürnberg (No.: 220_20 B).

**Table 1:** Sample characteristics

			Relationship quality			
Variables	Cohort M (SD) or n (%)	Negative/neutral M (SD) or n (%) *n* = 408 (42.4)		Positive M (SD) or n (%) *n* = 554 (57.6)	Cohen’s d	p^a, b,c^
**Caregiver**						
Age (years)	62.0 (12.6)	63.0 (11.9)		61.3 (13.2)	0.14	**.036** ^**b**^
Gender (male)	235 (24.4)	95 (23.3)		140 (25.3)	NA	.479^c^
Employment (yes)	462 (48.0)	185 (45.3)		277 (50.0)	NA	.153^c^
Education					NA	.182^c^
No degree	5 (0.5)	0 (0.0)		5 (0.9)		
Secondary school	365 (37.9)	166 (40.7)		199 (35.9)		
High school	407 (42.3)	170 (41.7)		237 (42.8)		
A-levels	89 (9.3)	36 (8.8)		53 (9.6)		
University	96 (10.0)	36 (8.8)		60 (10.8)		
Relationship (spouses, yes)	291 (30.2)	135 (33.1)		156 (28.2)	NA	.100^c^
Subjective care burden^d^	16.7 (7.5)	19.5 (6.7)		14.7 (7.4)	0.68	**< .001** ^**b**^
Positive aspects^e^	17.4 (9.2)	14.7 (8.2)		19.4 (9.3)	-0.54	**< .001** ^**b**^
Dysfunctional coping^f, g^	6.2^g^ (1.6)	6.0^g^ (1.5)		6.3^g^ (1.6)	-0.20	**.003** ^**b**^
Emotion-focused coping^f^	3.7 (2.2)	3.7 (2.2)		3.7 (2.3)	-0.002	.979^b^
Problem-focused coping^f^	4.1 (2.0)	4.2 (2.0)		4.0 (1.9)	0.09	.185^b^
Intrinsic care motivation (yes)^g^	459 (47.7)	119 (29.2)		340 (61.4)	NA	**< .001** ^**c**^
Extrinsic care motivation (yes)^g^	322 (33.5)	186 (45.6)		136 (24.5)	NA	**< .001** ^**c**^
**Care recipient**						
Age (years)	82.1 (7.1)	81.9 (7.3)		82.3 (6.8)	-0.05	.471^b^
Gender (male)	317 (33.0)	147 (36.0)		170 (30.7)	NA	.081^c^
Dementia (yes)^i^	365 (37.9)	185 (45.3)		180 (32.5)	NA	**< .001** ^**c**^
Level of care^j^	1.9 (1.3)	2.0 (1.3)		1.9 (1.3)	0.13	**.045** ^**b**^
**Care situation**						
Living together (yes)	508 (52.8)	227 (55.6)		281 (50.7)	NA	.131^c^
Care duration (months)	49.0 (80.1)	51.7 (85.3)		47.0 (76.1)	0.06	.370^b^
Caring for several people (yes)	63 (6.5)	22 (5.4)		41 (7.4)	NA	.213^c^
ADLs (h/d)	8.9 (5.1)	9.2 (5.2)		8.6 (5.1)	0.11	.081^b^
Support from relatives or friends (yes)	578 (60.1)	241 (59.1)		337 (60.8)	NA	.581^c^
Informal help (number)	2.0 (1.9)	2.1 (1.9)		1.9 (1.9)	0.13	**.044** ^**b**^

*NotesN* = 962; ADLs: activities of daily living, sum of 3 items; NA: not applicable

^a^*p* < 0.05 printed in bold

^b^t-test for metric variables

^c^Pearson Chi-square test

^d^Subjective care burden measured with the Burden Scale for Family Caregivers (BSFC-s; Graessel et al., 2014), range 0–30

^e^Positive aspects of caregiving measured with the Positive Aspects of Caregiving scale (PAC; Tarlow et al., 2004), range 0–40

^f^Dysfunctional, emotion-focused, problem-focused coping: measured with two items, each subscale from the Brief COPE (Carver, 1997), range 0–8

^g^The item has been inverted, and higher values refer to a less often usage of this coping style

^h^Measured with the question “What is your main reason for caregiving at home?” The answer category “I give care because of my attachment to the person I care for” was used as a measure of intrinsic care motivation, and “I give care because I feel obligated to do so for the person I care for” was used as a measure of extrinsic care motivation (Graessel, 2000)

^i^Cause of care is dementia

^j^Level of care, range 0–5

### Materials

All variables of interest were assessed by means of self-report questionnaires that were filled out by the CGs. The questionnaires included the assessment of RQ via the item we developed as well as variables associated with the CG, the CR, and the care situation.

#### Relationship quality

**Fig. 1:**
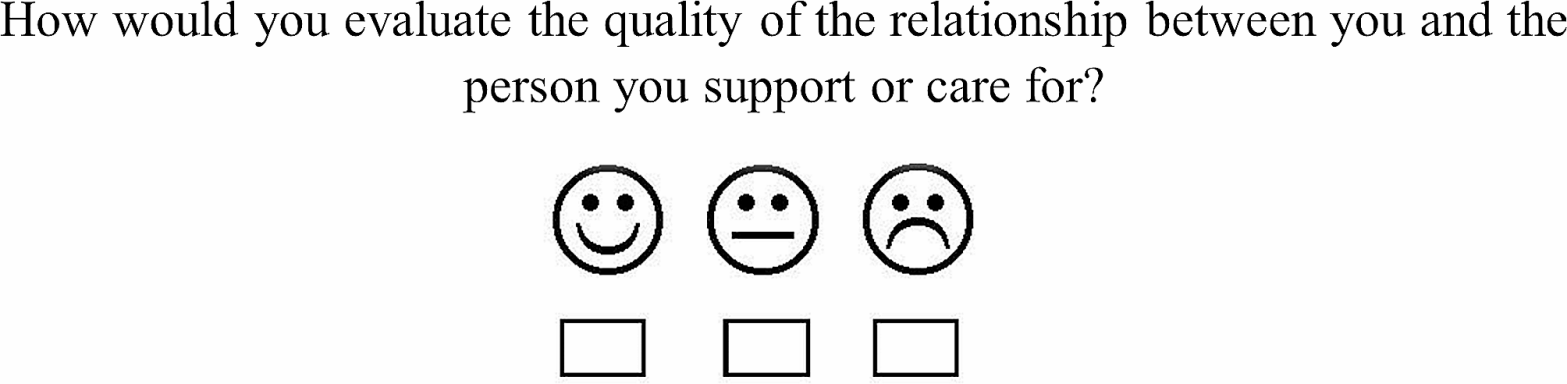
Item that was used to assess the quality of the relationship between the CG and CR. “How would you evaluate the quality of the relationship between you and the person you support or care for? [Wie schätzen Sie aktuell die Qualität der Beziehung zu der von Ihnen unterstützten, betreuten oder gepflegten Person ein? ]” For the analyses and to reduce the influence of social desirability, the answer options were dichotomized, and the neutral and negative categories were combined

We used our new item to assess RQ (Fig. 1). The simple visual representation of the answer options via smileys, characterizing the RQ as “positive,” “neutral,” or “negative” encourages non-vocal, honest, and spontaneous responses in CGs and can be easily implemented in a counseling setting. The visual representation of our answer options makes it independent of only one word (i.e., one adjective) as in the case of typical language-based scales. To find a symbol, which is (unconsciously) related to a collection of several adjectives which are related to emotional experiences, was our main idea behind using the smiley faces.

The item was “How would you evaluate the quality of the relationship between you and the person you support or care for?” The answer options were three smileys, one with a happy face, one neutral, and one with a sad face. The smileys were assigned to “positive,” “neutral,” and “negative” RQ. For the analyses, the answers were dichotomized by combining the categories “neutral” and “negative”. The reason for dichotomization was to overcome bias due to social desirability and to achieve similar group sizes.

#### Perceived burden

Perceived care burden was assessed with the Burden Scale for Family Caregivers-short version (BSFC-s; [38, 39]). Answers are given on a 4-point scale (0 = strongly disagree to 3 = strongly agree). Higher BSFC-s scores are associated with a stronger subjective burden for the CG. Cronbach’s alpha was 0.92 for the BSFC-s scale in our study.

#### Positive aspects

The perception of positive aspects of caregiving was assessed with the Positive Aspects of Caregiving scale (PAC; [40]). The PAC includes two parts: (1) self-affirmation and (2) life perspective. Items are rated on a 5-point scale (0 = fully applies to 4 = does not apply at all). Cronbach’s alpha was 0.93 for the PAC in our study.

**Care motivation**. Motivation for caregiving was assessed with the question “What is your main reason for providing caregiving at home?” Seven answer options were provided, of which one was “I give care because of my attachment to the person I care for” and another “I give care because I feel obligated to do so for the person I care for” [41]. The former item was used as a measure of intrinsic care motivation and the latter as an indicator of extrinsic care motivation.

#### Coping

CGs´ general coping behavior was measured with a 6-item version of the Brief COPE questionnaire [42]. The Brief COPE measures the dimensions dysfunctional coping as well as emotion-focused and problem-focused coping, each of which is assessed with two items. Ratings are given on 5-point scales, ranging from 0 (strongly disagree) to 4 (strongly agree). Cronbach’s alphas were 0.18 for the dysfunctional coping, 0.77 for the emotion-focused, and 0.48 for the problem-focused sub-scales in our study. Because Cronbach’s alpha is not well-suited for 2-item scales, the Spearman’s Brown correlation coefficient Rho was additionally calculated as measure for internal consistency [43]. These were Rho = 0.01 for the dysfunctional coping, Rho = 0.63 for the emotion-focused, and Rho = 0.31 for the problem-focused sub-scales.

#### Sociodemographic variables

Sociodemographic information included CGs’ and CRs’ age and gender, CGs’ employment status, CGs’ highest level of education, the status of the relationship between the CG and CR (whether they were spouses or not), the medical condition that resulted in the need for care, as well as the number of CRs the CG was responsible for. The medical condition that resulted in the need for care was assessed with one item with several answer options, of which one was whether the CR was diagnosed with dementia. With regard to the current care situation, we also measured the duration of care, the living situation of the CG and CR, and the total time spent giving care per day for activities of daily living (ADLs; 44). Furthermore, we assessed whether the CG received support from relatives or friends by using one dichotomous item. Moreover, we assessed whether informal help was used. For this, a list of 15 informal offers of help, such as the Mobile Nursing Service (‘Ambulanter Pflegedienst’) or family counseling, was used. The total number of offers of help that were accepted was used in the analyses.

### Statistical analyses

For statistical analyses, IBM SPSS statistics (version 28 for Windows) was used. Descriptive statistics, including frequencies, means (M), and standard deviations (SD) were computed to describe the sample characteristics. Independent t-tests for metric variables and Pearson Chi-square tests for nominal and ordinal variables were used to test for group differences between the neutral/negative and positive RQ groups.

To evaluate the determinants associated with our item, we computed a binary logistic regression with the dichotomized measure of RQ (0 = neutral/negative and 1 = positive) as the dependent variable. The regression was conducted in three blocks. In Block 1, the variables CGs’ age, gender, and education were included by using the enter method for controlling for these covariates. In Block 2, the variables burden, benefits, as well as extrinsic and intrinsic motivation, which refer to our hypotheses, were included by using forward selection. In Block 3, all other variables that did not show multicollinearity were also included by using forward selection. If the predictors exhibited multicollinearity, that is, if r (Pearson) or Spearman ρ > 0.60, the predictor with the higher bivariate correlation with the outcome was included in the regression model. The significance level was set at *p* < .01 because of the large number of cases, and p values less than 0.05 and greater than or equal to 0.01 were considered as statistical trends. Thus, the threshold for the inclusion of a predictor was *p* = .05. The criterion for removal was *p* = .10.

## Results

### Descriptive statistics

Descriptive statistics for the sample characteristics are provided in Table 1. Of the *N* = 962 CGs, *n* = 554 (57.6%) reported having a positive relationship and *n* = 408 (42.4%) reported a negative or neutral relationship (*n* = 359 [37.3%] neutral and *n* = 49 [5.1%] negative) with the CR. CGs who reported a neutral or negative RQ were significantly older (*p* = .036), reported higher burden (*p* < .001), less positive aspects of caregiving (*p* < .001), used dysfunctional coping less often (*p* = .003), and reported more often extrinsic (*p* < .001) and rarer intrinsic care motivation (*p* < .001) than the positive relationship group. In the negative/neutral group, more CRs were diagnosed with dementia (*p* < .001), and the care level was higher (*p* = .045) than in the positive group. Furthermore, the number of CGs who used informal help was higher in the negative/neutral group than in the positive group (*p* = .044).

### Binary logistic regression

An analysis of multicollinearity revealed a significant association between the variables employment status and CGs’ age (ρ = 0.68, *p* < .001) as well as between relationship status and living situation (ρ = 0.61, *p* < .001). Due to higher correlations with RQ, the variables age and relationship status were included in the binary logistic regression. Due to lower correlations with RQ, the variables employment status and living situation were excluded.

The binary logistic regression analysis (Table 2) revealed a significant model (χ² = 279.18, *p* < .001) with the six significant predictors CGs’ and CRs’ age as well as subjective burden, perceptions of positive aspects, as well as intrinsic and extrinsic care motivation. Older CGs reported lower RQ (B = -0.39, *p* < .001). More perceived care burden as well as a higher amount of extrinsic care motivation predicted a lower RQ (burden: B = -0.07, *p* < .001; extrinsic motivation: B = -0.45, *p* = .007). More perceived positive aspects as well as a higher amount of intrinsic care motivation predicted a higher RQ (positive aspects: B = 0.07, *p* < .001; intrinsic motivation: B = 1.19, *p* < .001). Furthermore, RQ was lower for younger CRs (B = 0.04, *p* = .004).

**Table 2:** Binary logistic regression for RQ as the dependent variable; model: enter (Block 1), forward selection (Blocks 2 and 3)

Predictor	Relationship quality			
	B	*p* ^a^	OR	95% CI (OR)
**Block 1** ^b^				
Age	-0.39	**< 0.001**	0.96	[0.94, 0.98]
Gender	0.18	0.351	1.20	[0.82, 1.76]
Education	0.06	0.506	1.06	[0.89, 1.26]
**Block 2** ^**c**^				
In equation: Subjective care burden^d^	-0.07	**< 0.001**	0.93	[0.91, 0.95]
Positive aspects^e^	0.07	**< 0.001**	1.07	[1.05, 1.09]
Intrinsic care motivation (yes)^f^	1.19	**< 0.001**	3.29	[2.37, 4.57]
Extrinsic care motivation (yes)^f^	-0.45	**0.007**	0.64	[0.46, 0.89]
**Block 3** ^**g**^				
In equation:				
Age CR	0.04	**0.004**	1.04	[1.01, 1.07]
Dementia (yes)	-0.34	0.036	0.71	[0.52, 0.98]
Dysfunctional coping^h, i^	0.12	0.015	1.13	[1.03, 1.25]
Caring for several people (yes)	0.72	0.028	2.06	[1.08, 3.91]
Not in equation:				
Gender CR	-0.10	0.594	0.90	[0.62, 1.32]
Level of care	-0.08	0.240	0.93	[0.81, 1.05]
Emotion-focused coping^h^	-0.03	0.416	0.97	[0.90, 1.05]
Problem-focused coping^h^	-0.06	0.202	0.94	[0.86, 1.03]
Relationship (spouses, yes)	-0.16	0.599	0.85	[0.47, 1.55]
Care duration (month)	0.00	0.746	1.00	[0.998, 1.002]
ADL (h/d)	0.03	0.115	1.03	[0.99, 1.06]
Support from relatives or friends (yes)	-0.10	0.532	0.90	[0.66, 1.24]
Informal help (number)	-0.04	0.326	0.96	[0.88, 1.04]

*NotesN* = 962; B = Non-standardized regression coefficient B; OR = Odds ratio; CI = Confidence interval; relationship quality: 0 = negative/neutral, 1 = positive; ADLs: activities of daily living

^a^*p* < 0.01 printed in bold

^b^Adjustment variables (covariates) using the enter method: variables relate only to CGs.

^c^Variables referring to our hypotheses using stepwise forward selection and *p* = .05

^d^Subjective care burden measured with the Burden Scale for Family Caregivers (BSFC-s; Graessel et al., 2014), range 0–30

^e^Positive aspects of caregiving measured with the Positive Aspects of Caregiving scale (PAC; Tarlow et al., 2004), range 0–40

^f^Measured with the question “What is your main reason for providing caregiving at home?” The answer category “I give care because of my attachment to the person I care for” was used as a measure of intrinsic care motivation, and “I give care because I feel obligated to do so for the person I care for” as a measure of extrinsic care motivation (Graessel, 2000)

^g^Variables referring to the exploratory analysis using stepwise forward selection and *p* = .01

^h^Dysfunctional, emotion-focused, problem-focused coping: measured with two items, each subscale from the Brief COPE (Carver, 1997), range 0–8

^i^The item has been inverted, and higher values refer to a less often usage of this coping style

Moreover, some additional trends (*p* < .05) were identified. These were CR’s diagnosis was dementia, CG’s dysfunctional coping, and whether the CG was caring for more than one CR. If the CR’s diagnosis was dementia, lower RQ was reported by the CG (B = -0.34, *p* = .036). Caregivers who were caring for more than one CR and caregivers who used less dysfunctional coping reported higher levels of RQ (more than 1 CRs: B = 0.72, *p* = .028; dysfunctional coping: B = 0.12, *p* = .015, note that this item has been inverted and higher values refer to a less often usage of this coping style). Nagelkerke’s R^2^ was 0.34, which indicates that the examined variables increased the amount of variance that was explained to 34%.

## Discussion

The aim of our study was to evaluate a newly developed single item that could be used to assess RQ in informal caregiving of older people in a very quick and non-verbal way. The hypothesized associations – all of which have been previously reported in numerous studies using other, more complex tools (e.g., [33, 45]) – with the variables perceived care burden, perceived positive aspects, as well as intrinsic and extrinsic care motivation were supported in favor of our tool. This confirms the construct validity of the proposed one item assessment of RQ. Overall, all of our hypotheses were supported, and the expected associations between RQ and subjective care burden, perceived positive aspects, and intrinsic as well as extrinsic care motivation were confirmed. Moreover, additional predictors were identified: CR’s age as well as whether the CR’s diagnosis was dementia, CG’s dysfunctional coping, and whether the CG was caring for more than one CR.

The most important advantage of our item is that it offers a very fast way to gain an overview of the current relationship between the CG and the CR, in contrast to previous questionnaires that included more items and were developed for other contexts [15–19]. In particular, the language-free, cross-cultural presentation of the answer options suits the target population, and the sensitive handling of social desirability by combining only the neutral and negative answer categories during the evaluation makes the application much more pleasant for the counselor and the CG. All these potential advantages, which should be evaluated in future studies, should make it easier to address and evaluate this sensitive topic in counseling settings.

Our findings support the literature-based associations between RQ and the variables perceived care burden, perceived positive aspects, and intrinsic as well as extrinsic care motivation. Perceived care burden has been found to be associated with the CG’s long-term mental and physical health and is therefore a key factor to maintain the care situation for as long as possible [10, 11, 14]. Extrinsic care motivation has also been found to be – besides with the RQ between CG and CR – associated with stress and subjective care burden [12, 34] and is, therefore, also a highly relevant risk factor. More positive aspects of caregiving are also related to a lower likelihood that the CR will be placed in a nursing home [36] and should therefore also come more in the focus of research in the context of informal caregiving [37].

Beside these key factors, we identified additional significant predictors of RQ. One was CR’s age, which was higher when RQ was more positive. A reason for this finding may be that older CGs have already been caring longer for the CR, which may be associated with a delay of placing the CR in a nursing home. Another predictor that was significant by trend was whether the CR’s diagnosis was dementia. CGs who cared for a CR with a dementia diagnosis more often reported a negative or neutral relationship [46]. One explanation for this finding is that dementia is often accompanied by demanding behavior, which tends to have a negative impact on RQ [47]. Furthermore, this finding is line with previous research, which showed that caring for people who are diagnosed with dementia, puts a high amount of burden on the CG (e.g., [48]), and that disease (i.e., dementia)-related factors are one of the most important predictors of CG’s burden for this target-group [49]. Moreover, we found that CGs who reported a smaller amount of dysfunctional coping, and therefore used an adaptive (in contrast to a maladaptive) coping style more often reported a positive CG-CR relationship. This finding fits well into research on relationship quality in romantic partnerships [50, 51] and suggests that interventions, that aim at improving individual coping behavior may also be suited to increase RQ.

Another potential predictor that we identified in our exploratory analysis was whether the CG was caring for several people. Caregivers who were involved in caregiving for more than one CR more often reported a positive relationship. This finding is in line with the general assumption that RQ is an important factor in informal caregiving because caring for several people is possible only when this situation is not associated with large amounts of stress or burden or additional negative factors. Providing care for more than one person can be endured only when the relationship is right and the situation is perceived as more positive than negative.

The use of our item is not restricted to researchers. It can also be used by general practitioners, nurses who provide home care, or further people who are involved in informal caregiving of older people. Moreover, the use of our item is – although this must be evaluated in future studies – not restricted to the context of informal caregiving of older people. Other possible areas of application are all settings, in which RQ in couples should be assessed (e.g., for assessing child-parent, friendship, or romantic RQ).

Our findings have some theoretical implications. The first one refers to the dichotomization. Our dichotomous evaluation is the simplest form of measuring variability, namely evaluating two expressions. Dichotomous characteristics do not only occur in numerous physical and technical systems (e.g., geomagnetism with north and south poles or binary coding in computers), but also in the living world. The biological distinction between the two sexes female and male is enough to produce a sufficient number of new combinations of genes. However, we explicitly do not want to discriminate against anyone and value the diversity of other gender identities. Further examples are signal transmission in neurons that either fire or not. Our findings suggest that dichotomization of RQ is not a too strong simplification, but rather the dichotomization is sufficient for a valid depiction of reality; at least as a first approach in order to make a further differentiation later if necessary.

The second one refers to the usage of pictograms (i.e., smiley faces). Pictograms can usually be grasped quickly, as they are used in many everyday applications. Prominent examples are traffic signs, icons in software, or the on/off-symbol on electronic devices [52]. The processing of simple and highly familiar visual information in the brain occurs quickly within a few hundred milliseconds [53, 54]. Familiar symbols are processed in early visual brain areas in a bottom-up way [55, 56]. Especially faces, or pictograms which mimic emotional facial expressions are in favor of fast processing [57, 58], which is associated with an evolutionary advantage [59]. In our study, we used such familiar, emotional smiley faces as answer categories for our item (i.e., one positive, friendly or happy one, one with a neutral emotional expression, as well as a negative, sad one). The used symbols should also be highly familiar and overlearned and are, thus, in favor of fast, effortless processing. Therefore, we conclude that assessing the psychological construct RQ by means of our item is achieved quickly and precisely, as well. This fast, unconscious processing (i.e., without a major influence of top-down processes) may be another reason that bias due to social desirability is reduced compared to language-based scales.

One limitation of our study pertains to the local sample of CG-CR couples from the Bavarian area in Germany. However, due to the language-free assessment, it is very likely that our findings can be generalized to other German and non-German areas. Furthermore, our study was limited to the context of informal caregiving of older people. Future research should be conducted in other contexts, such as non-geriatric informal caregiving or in nursing homes. In addition, our one item RQ assessment should be evaluated in contexts that are not related to care situations. It is well-known that RQ in CG-CR relationships is lower than for non-caregiving control couples [23]. CGs gave significantly lower RQ ratings than controls.

Beside the advantage of decreasing social desirability bias, the dichotomization has also the drawback that information gets lost. Furthermore, it is uncertain to what extent the dichotomization actually compensated social desirability bias in the current study, because respondents who gave socially desirable answers may have answered the item with “positive” rather than with “neutral”. Nevertheless, our approach (i.e., combining the “negative” and “neutral” category) is better than not correcting for social desirability at all and our results indicate that we were – at least partially – successful.

Moreover, associations with additional factors should be investigated in future studies. One of these is the meaning that CGs derive from their role as CG, which has been found to be related to intrinsic care motivation as well as to CGs’ well-being [60]. Furthermore, specific reasons for CGs’ burden (e.g., the burden related to the care situation vs. the burden related to other social networks; [61]) and their associations with RQ should be addressed in future research. Here, a further direction is to assess the user friendliness of our new item (i.e., the CR’s perception and usability of the item) and to compare its handling with existing tools. Mixed-method approaches, in which qualitative and quantitative methods are combined, may be well suited to reach this goal.

Moreover, it should be noted that although one item can be easy and fast to apply in practice, this can also bring some problems, because it is not multi-faceted, and some information can get lost. For example, users get no information on what exactly is satisfactory or poor in the relationship. Using additional questions or combining our tool with qualitative approaches would enable evaluating what is going well or not in the relationship. However, using one item can have more advantages than drawbacks in many cases, especially in the context of informal caregiving of older people.

Our study has a great deal of relevance due to the increasing numbers of informal caregiving situations in recent years [62–64]. Besides reducing stress and other negative factors associated with the care situation (e.g., [65]), interventions should focus on improving or maintaining positive aspects of the care situation [4, 66], one of which is a positive relationship between the CG and CR [5, 6]. Our tool can be used to evaluate these interventions. It has the potential – although this still needs to be evaluated – to be easily applied at multiple assessment time points without putting a great burden on the CG.

## Conclusions

In this study, we presented a new item with which RQ can be easily assessed in the context of informal caregiving of older people by minimizing the influence of social desirability. We were able to confirm the associations between RQ and the variables perceived care burden, perceived positive aspects, as well as intrinsic and extrinsic care motivation, all of which have been previously reported. Overall, our study highlights the importance of considering RQ in research on informal caregiving. Our findings offer starting points for the development of interventions to improve the care situation and CGs’ health and well-being. Our item has the potential to be used in all settings, in which RQ in couples should be assessed.

## Data Availability

The data sets used and analyzed for the current study are available from the corresponding author upon reasonable request.

## Abbreviations

ADLs: Activities of daily living
BSFC-s: Burden scale for family caregivers – short version
CG: Caregiver
CR: Care recipient
MD: Bavaria medical service of the bavarian health insurance
PAC: Positive aspects of caregiving
RQ: Relationship quality
SHI: Statutory health and nursing care insurance
